# Decomposing mosaic tandem repeats accurately from long reads

**DOI:** 10.1093/bioinformatics/btad185

**Published:** 2023-04-11

**Authors:** Bansho Masutani, Riki Kawahara, Shinichi Morishita

**Affiliations:** Department of Computational Biology and Medical Sciences, Graduate School of Frontier Sciences, The University of Tokyo, Chiba 277-8562, Japan

## Abstract

**Motivation:**

Over the past 30 years, extended tandem repeats (TRs) have been correlated with ∼60 diseases with high odds ratios, and most known TRs consist of single repeat units. However, in the last few years, mosaic TRs composed of different units have been found to be associated with several brain disorders by long-read sequencing techniques. Mosaic TRs are difficult-to-characterize sequence configurations that are usually confirmed by manual inspection. Widely used tools are not designed to solve the mosaic TR problem and often fail to properly decompose mosaic TRs.

**Results:**

We propose an efficient algorithm that can decompose mosaic TRs in the input string with high sensitivity. Using synthetic benchmark data, we demonstrate that our program named uTR outperforms TRF and RepeatMasker in terms of prediction accuracy, this is especially true when mosaic TRs are more complex, and uTR is faster than TRF and RepeatMasker in most cases.

**Availability and implementation:**

The software program uTR that implements the proposed algorithm is available at https://github.com/morisUtokyo/uTR.

## 1 Introduction

Tandem repeats (TRs) are consecutive genomic sequence duplications of one or more units in tandem (Smith 1976). In the early 1980s, when TRs consisting of units of 2–6 base pairs (bp) were discovered, they were called microsatellites (Miesfeld et al. 1981; Spritz 1981; Hamada and Kakunaga 1982) but were later referred to as short sequence repeats, short tandem repeats, or simple repeats (Ellegren 2004). In 1985, TRs of units of a few dozen base pairs were also discovered and designated as minisatellites to distinguish them from microsatellites (Jeffreys et al. 1985). Micro- and minisatellites varying in length among individuals were identified (Tautz et al. 1986), and referred to as variable number tandem repeats (VNTR), which have been valuable for understanding genetic diversity in human populations (Bowcock et al. 1994; Weber and Wong 1993). Here, micro-/minisatellites and VNTRs are collectively referred to as TRs.

With the availability of abundant genomic data, such as individual exome and whole genome sequences on a population basis, a number of algorithms have been proposed to estimate the length and structure of TRs on a genome-wide basis in individual genomes (Dashnow et al. 2018; Dolzhenko et al. 2019, 2020; Mousavi et al. 2019), although it is still difficult to accurately determine the entire structure of TRs of >100 bp in length using short-read sequencing. Therefore, long-read sequencing platforms, such as PacBio and Nanopore, have recently become attractive because long-read sequencing can cover most TRs <10 kb in length and can sequence long DNA fragments without using polymerase chain reaction (PCR), which is prone to replication slippages during amplification (Hannan 2018). An initial study of using long read sequencing suggested that ∼30% of structural variants are TRs (Audano et al. 2019).

Over the past 30 years since 1990, ∼60 diseases have been shown to be associated with extremely expanded TRs at different loci, which are assumed to be disease-causing variants (Depienne and Mandel 2021). The diversity of TRs is considered to provide insight into missing heritability. Long-read sequencing is expected to uncover two types of hidden, disease-associated TRs: minisatellites and mosaic TRs. Indeed, several disease-associated minisatellites have been identified (Cortese et al. 2019; Course et al. 2020; De Roeck et al. 2018; Song et al. 2018).

Another type of disease-associated TR has a mosaic structure in which different units are expanded in individual genomes. For example, a total of 400–2000 copies of AAAAG, AAAGG, AAGAG, and AGAGC within the RFC1 gene are associated with cerebellar ataxia, neuropathy, vestibular areflexia syndrome (CANVAS) (Cortese et al. 2019). Several disease-associated mosaic TRs have been reported to date (Ishiura et al. 2018; Koob et al. 1999; Liquori et al. 2001; Wright et al. 2019). The sequence compositions of mosaic TRs are difficult to characterize and are usually confirmed by manual inspection. Widely used tools such as PBSV, TRF (Benson 1999), and RepeatMasker (http://repeatmasker.org) were not designed to compensate for this issue and often fail to correctly parse mosaic TRs. For example, repeats of the form (AAAG)*i* (AG)*j* (AGGG)*k* (AG)*l* (AAAG)*m*, where the italic suffixes indicate the number of unit occurrences, are likely to be falsely detected as a single (AAAG)-repeat or (AG)-repeat. Therefore, we developed computational algorithms to address the issue of automatic characterization of mosaics in TRs.

## 2 Materials and methods

### 2.1 Approximate regular expression matching problem

Replication slippage and/or non-homologous recombination are thought to drive TR stretching that generates tandem copies of units. For this reason, mosaic TRs often have a limited number of units specific to them. It is crucial to find a set of strings (units) *U* for the input string (TR) *S*.

Before proposing a method for selecting the unit set *U*, we point out here that once *U* is selected, an optimal solution can be obtained according to a reasonable measure with a known efficient algorithm. The idea is to calculate an optimal concatenation of elements in *U* that partially matches the input *S* with the minimum Levenshtein distance (i.e. the sum of substitutions, insertions, and deletions) of the global alignment between *S* and the concatenation. Partial matches are taken into account to accommodate sequence errors and unit-copy mutations. This is a subproblem of the approximate regular expression matching problem, which is to calculate an optimal instance of a regular expression that maximizes its alignment score with a given string, because a concatenation of elements in *U* is represented as the regular expression $(u_{1}|\ldots |u_{k})*$ for all units $u_{1},\ldots ,u_{k}$ in *U*. The approximate regular expression matching problem can be solved efficiently in $O(|S|\cdot \sum_{u\in U}|u|)$ (Myers and Miller 1989). This concept has recently been reinvented and used in the string decomposer algorithm (Dvorkina et al. 2020), which is a wraparound dynamic programming algorithm that handles a set of multiple repeat units, say *U*. In the next subsection, we propose a method for selecting a better set of units.

### 2.2 Selecting a better set of units according to maximum parsimony that minimizes replication slippage events

We continue to use *S* and *U* for denoting a string of length *n* and a set of substrings in *S* that respectively represent a TR and units in it. Suppose that *S* is decomposed into a series of neighboring, non-overlapping substrings $S[b_{i},e_{i})$ for i=1,…,k such that $S[b_{i},e_{i})$ begins at *b_i_* and ends at $e_{i}-1,\;b_{1}=0,\;e_{i}=b_{i+1}(i<k)$, *e_k_* = *n*, and all $S[b_{i},e_{i})$ are present in *U*. Such a series is called a *decomposition D* of *S* by *U*. There could be more than one decomposition of *S* by *U*. For example, when *S* = ACCGACCGACCG and *U* = {ACCG, AC, CG, CGAC}, consider the following decompositions of *S* by *U*:


$$D_{1}=\mathrm{ACCG}\;\mathrm{ACCG}\;\mathrm{ACCG}\;\;D_{2}=\mathrm{AC}\;\mathrm{CG}\;\mathrm{AC}\;\mathrm{CG}\;\mathrm{AC}\;\mathrm{CG}\;\;D_{3}=\mathrm{AC}\;\mathrm{CGAC}\;\mathrm{CGAC}\;\mathrm{CG}$$

To measure the goodness of decomposition *D* of *S* by *U*, assuming maximum parsimony that prefers fewer events of replicate slippage and/or non-homologous recombination, we design the penalty of *D* to be smaller when *D* consists of fewer copies of fewer and shorter generating units. For this purpose, we associate each unit u∈U with the *penalty*, |u|+o(u), where *o*(*u*) is the number of occurrences of *u* in *D* (Dvorkina et al. 2021), and set the penalty of *D* to the sum of penalties, $\sum_{u\in U}(|u|+o(u))$. In the running example, the penalty of *D*_1_ is 15=(4 + 3)+(2 + 0)+(2 + 0)+(4 + 0) because ACCG appears three times in tandem, while no other unit of *U* appears. Similarly, the penalties of *D*_2_ and *D*_3_ are 18 and 16, showing that *D*_1_ has the minimum penalty. For a given decomposition, *U* can be made smaller by eliminating unnecessary elements from *U* that do not appear in the decomposition: for example, by setting *U* = {ACCG} for *D*_1_, the penalty of *D*_1_ becomes 7(=4 + 3).

In general, a variety of unit sets can be candidates. Extreme examples are the set of all single letters and the singleton set of the entire string *S*. The penalty of the former set is k+|S| when the number of letters is *k*, the penalty of the latter is |S|+1, and both penalties are large. Extremely long or short units can generally be avoided by selecting a pair of *U* and *D* for *S* that minimizes the penalty $\sum_{u\in U}(|u|+o(u))$.

In practice, we had to generalize the penalty function to accommodate sequencing errors. Sequencing errors often generate infrequent units in a TR. Such rarely occurring outlier units can be placed in *U*, but may unnecessarily enlarge the unit set *U* with rare units. Instead, we took the approach of excluding these rare units. To this end, we measure the degree that *most* substrings are in *U*, and we define the *coverage* of *D* by *U* as the total length of substrings of *D* that are also present in *U*; i.e. $\sum_{s\in D,s\in U}|s|.$ Thus, it is ideal to find *U* that minimizes the penalty of *D* by *U*, and maximizes the coverage of *D* by *U*; however, it may not always be possible to optimize both of these criteria simultaneously. Of note, as the latter maximization is equivalent to the minimization of $\sum_{s\in D,s\notin U}|s|\;\;(=|S|-\sum_{s\in D,s\in U}|s|)$, we instead attempt to minimize: in order to output nearly optimal values of the penalty and coverage of *D* by *U*. Therefore, we generalize the penalty of *D* by *U* to the above formula, and call it the *extended* penalty.


$$\sum_{u\in U}(|u|+o(u))+\sum_{s\in D,s\notin U}|s|$$

Whether it is intractable to compute a pair of *U* and *D* that minimizes the extended penalty is an open question. We have considered this problem, but with partial results, which will be discussed later. Because of this situation, we implemented a greedy algorithm that initially assigns the empty set to *U* and lets decomposition *D* undivided. It then repeats the process of selecting and adding to *U* the best unit that minimizes the extended penalty defined above until no more units can be selected to minimize the extended penalty. Using the running example, we illustrate how the greedy algorithm works. For input *S*, set *U* to the empty set:


U={}   S=ACCGACCGACCG

We can select more than one unit as the first candidate (e.g. ACCG, AC, CG, and CGAC). If we select and add ACCG to *U*, we have decomposition *D*_1_:


$$U=\{\mathrm{ACCG}\}\;\;\;D_{1}=\mathrm{ACCG}\;\mathrm{ACCG}\;\mathrm{ACCG}$$

Because ACCG ∈ *U* is of length 4 and has 3 occurrences, the extended penalty becomes 7 = 4 + 3. Instead, if we select AC,


$$U=\{\mathrm{AC}\}\;\;\;D_{2}=\mathrm{AC}\;\mathrm{CG}\;\mathrm{AC}\;\mathrm{CG}\;\mathrm{AC}\;\mathrm{CG}$$

AC ∈ *U* is of length 2 and has 3 occurrences, but 3 instances of CG are absent in *U*, and the extended penalty is 11=2+3+2×3. Selecting CG instead also outputs the penalty of 11. Selection of CGAC yields:


$$U=\{\mathrm{CGAC}\}\;\;\;D_{3}=\mathrm{AC}\;\mathrm{CGAC}\;\mathrm{CGAC}\;\mathrm{CG}$$

The penalty is 10=4+2+2+2 as *U* excludes AC and CG. Thus, ACCG minimizes the penalty and covers *D*_1_ entirely. No more units are selected.

HORDecomposer has been proposed to detect higher-order repeats (HORs), a special case of centromere mosaic TRs, from a series of alpha satellite monomers of length ∼171 in human centromeres (Dvorkina et al. 2021). Our algorithm differs from HORDecomposer in that it used the extended penalty function defined above, and it focuses on accelerating the detection of candidate shorter units of various lengths ≤100 b using suffix arrays and Burrow–Wheeler transform. We will show that this greedy algorithm can estimate benchmark mosaic TRs with high accuracy in the section of experimental results.

### 2.3 Mosaic tandem repeats

Here, we formally define “mosaic tandem repeats.” A *tandem repeat* of string *u* is defined as having the form $s{\left(u\right)}^{k}p$ such that *s* and *p* are a suffix and a prefix of *u*, respectively, and ${\left(u\right)}^{k}$ is a series of copies of *u*. To eliminate ambiguity, assume that *u* is not a TR of any shorter substring, and a TR of *u* is maximal such that none of its superstrings are TRs of *u*. Decomposition *D* is *mosaic tandem repeats* of *U* if and only if the coverage of *D* by TRs of units in *U*,

∑{|s||s∈D is a substring of a TR of unit u∈U}exceeds a given threshold (e.g. |S|×0.8). For example, when *U* = {AC, CGA}, for example,

- AC AC C CGA CGA is mosaic TRs of *U*, but
- AC CGA AC T CGA is not if the threshold is set to |S|×0.8.

We can select more than one unit from a TR of a single unit. For example, in (AAGA)^*k*^, any rotation of AAGA can be a unit because and AAG and A are a suffix and a prefix of AAAG, respectively. To resolve ambiguity and select a representative unit, we define that a substring is *self-overlapping* if a proper suffix matches a prefix; otherwise, it is *non-self-overlapping*. There exists a non-self-overlapping unit because any unit is not a TR. In the running example, AAAG and GAAA are non-self-overlapping units. In the next subsection, we present the design of an $\Theta (n^{2})$-time algorithm for enumerating all non-self-overlapping substrings in string *S* of size *n*.


$${\left(\mathrm{AAGA}\right)}^{k}=\mathrm{AAG}{\left(\mathrm{AAAG}\right)}^{k-1}A,$$

### 2.4 Algorithm for enumerating repetitive non-self-overlapping substrings

In this section, we introduce an algorithm for efficiently enumerating repetitive non-self-overlapping substrings of given string *S* of length *n*. We determine for each (i,j) whether S[i,j) is repetitive and whether S[i,j) is non-self-overlapping respectively, and add it to the list if both are satisfied.

First, we describe how to determine if S[i,j) is repetitive. For this purpose, we define an array *LEN* as follows:


LEN[i]=max{l|S[i,i+l) appears twice or more in S}

If and only if LEN[i]≥j−i, S[i,j) is repetitive. If we denote the length of the longest common prefix (LCP) of S[i,n) and S[j,n) as lcp(i,j), we have LEN[i]=max{lcp(i,j)|j≠i}. The closer *i* and *j* are on the suffix array of *S*, the larger lcp(i,j) becomes. Therefore, for the two (or one) suffixes adjacent to S[i,n) on the suffix array, we calculate the length of the longest common suffix with S[i,n), and the maximum of these is the value of LEN[i]. Given a suffix array and LCP array of *S*, this can be computed in *O*(*n*) (see Algorithm 1). We can obtain the suffix array using SA-IS (Nong et al. 2009), and the LCP array using Kasai’s algorithm (Kasai et al. 2001), both in *O*(*n*).

Algorithm 1 Calculate maximum lengths of repetitive substring starting with *i* for each *i*

**Input:** String *S*

**Output:** Array of maximum lengths of repetitive substrings *LEN*

1: **function** CALC_LEN(*S*)

2: n←|S|

3: Prepare an array *LEN* of length *n* and initialize all elements to 0.

4: SA← suffix array of *S*

5: LCP← longest common prefix array of *S*

6: $\mathrm{LEN}[SA[i]]\leftarrow LCP_{1}$

7: **for**i=2…|S|: $\mathrm{LEN}[SA[i]]\leftarrow \max(LCP_{i-1},LCP_{i})$

8: **return** *LEN*

Second, we describe how to determine if S[i,j) is non-self-overlapping. For each *i*, let *OL_i_* be the array of length *n* − *i*:


$$OL_{i}[k]=\max\{l|S[i,i+l)=S[i+k-l,i+k)\}$$

If and only if $OL_{i}[j-i]=0,\;S[i,j)$ is non-self-overlapping. Construction of *OL_i_* can be performed in O(n−i) time, using the Morris-Pratt algorithm (Morris and Pratt 1970). Combining the above two algorithms, we can enumerate repetitive non-self-overlapping substrings (see Algorithm 2). In Algorithm 2, Line 5 calling the O(n−i) process for each *i* is the bottleneck, and the overall complexity is $O(n^{2})$.

Algorithm 2 Enumaration of repetitive non-self-overlapping substrings

**Input:** String *S*

**Output:** List of repetitive non-self-overlapping substrings *L*

1: n←|S|

2: L←∅

3: LEN← CALC_LEN(*S*)

4: **for**i=1…n:

5:  $OL_{i}\leftarrow$ MORRIS_PRATT(S[i,n))

6:  **for**j=2…n+1:

7:  **if**LEN[i]≥j−i and $OL_{i}[j-i]=0$:

8:      L←L∪{(i,j)}

9: **return** *L*

### 2.5 Algorithm for decomposing an input string

To decompose an input string *S* into mosaic TRs efficiently, we are now in a position to combine the algorithms presented in this section:

1. With Algorithm 2, calculate the set of repetitive non-self-overlapping substrings in *S* and assign the set to *V*.
2. Set *U* to the empty set. Repeat the process that selects the best unit from *V* with the minimum extended penalty and adds the unit into *U* until no more units can be selected to minimize the penalty.
3. Compute an optimal concatenation of elements in *U* that partially matches the input *S*. For this purpose, an $O(|S|\cdot \sum_{u\in U}|u|)$-time algorithm for solving the approximate regular expression matching problem (Myers and Miller 1989) or the string decomposer (Dvorkina et al. 2020), a wraparound dynamic programming algorithm, are available.

## 3 Results

### 3.1 Accuracy of decomposition

To demonstrate that the program, named uTR, can decompose various mosaic TRs, we created a test data set consisting of typical mosaic TRs with widely different units:

- (AC) *i* (AG) *j*(ACC) *i* (GTT) *j*(AAG) *i* (AG) *j*(AAG) *i*(AGG) *j*
- (AAAG) *i*(AG) *j*(AAAG) *i* (AG) *j* (AAAG) *k*
- (AAAG) *i*(AG) *j*(AGGG) *k*(AG) *l*(AAAG) *m*
- (AAAAAG) *i*(AAAGAGAGGGAAAAG) *j*(AGGGG) *k*

The variable (e.g. i,j,k,l,m) next to each unit in parentheses represents the number of unit occurrences. Mosaic TRs are harder to decompose correctly when more distinct units are present, and different units are more similar. To understand the hardness of the decomposition, we generate a variety of datasets of different lengths for each mosaic TR pattern; i.e. variables in each pattern were set to random values ranging from 10 to 200.

To see how sequencing errors affect the prediction of the original mosaic TR patterns, letters of strings in each dataset were modified at random by sequencing errors (substitutions, insertions, and deletions) at the rate of 0%, 1%, 3%, 5%, 10%, and 15%. When a mosaic TR has three units ${\left(U\right)}_{i}{\left(V\right)}_{j}{\left(W\right)}_{k}$, for example, all of the three units need to be predicted nearly accurately. A series of units ${\left(U\right)}_{i}$ is accurate if the value of *i* differs by at most X% of the true value, where we call X% an *allowance* and set X% to 0%, 1%, 2%,…, for example. Accuracy increases by setting allowance X% to a larger value, and this mitigation is reasonable and necessary when dealing with two homologous units (i.e. AAG and AG) because it becomes ambiguous to correctly determine the boundary between two similar units in the presence of sequencing errors.

We compared the prediction accuracy of uTR with TRF (Version 4.09) and RepeatMasker (version open-4.0.7). We used RepeatMasker with default parameter settings (-e hmmer -noint -pa 4 -div 0 -xsmall) and TRF with default parameter settings except for lowering the minimum alignment score from 50 to 10 (i.e. 2 7 7 80 10 10 1000 -h -ngs) in order to detect small TRs with ≥10 units in our benchmark datasets. TRF sometimes returns a single most likely mosaic TR, but it often outputs a number of TRs some of which overlap each other. To find a mosaic TR, a series of non-overlapping TRs has to be selected, which is actually solved by RepeatMasker. Therefore, RepeatMasker seems to be better suited to detect mosaic TRs than TRFs, and this tendency will be confirmed empirically later.

**Figure 1.**
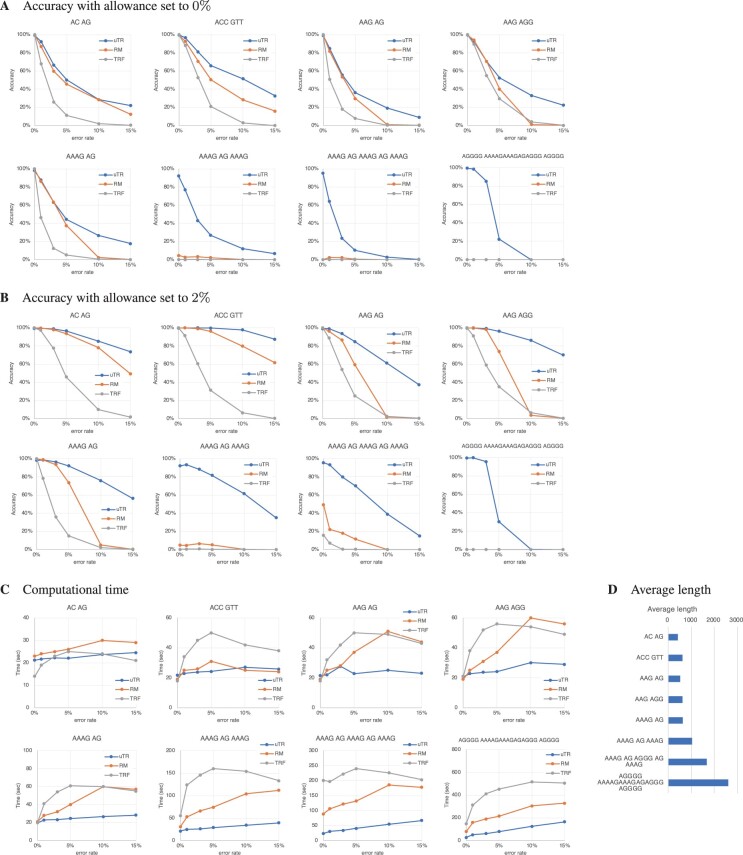
(A) Graphs show accuracy of eight mosaic TR patterns shown in the titles and of six sequencing error rates (0%, 1%, 3%, 5%, 10%, and 15%) in the *x*-axis when the allowance is set to 0%. The *y*-axis shows the prediction accuracy of uTR (blue), RepeatMasker (orange) and TRF (gray) to estimate the original patterns of 1000 strings. (B) Accuracy when the allowance is set to 2%. (C) The *y*-axis shows the total computation time (in seconds) when 48 datasets are processed by uTR, RepeatMasker, and TRF. The *x*-axis and graph legend are the same as Figure A. (D) The average length of 1000 mosaic TRs in each dataset. The test code is available at https://github.com/morisUtokyo/uTR/tree/main/test_public.

For each of eight mosaic TR patterns, we considered six sequencing error rates, and created a total of 48 (=8×6) datasets with 1000 strings. Appling uTR, RepeatMasker, and TRF to the datasets, we observed trends in prediction accuracy (Fig. 1A and 1B) when using different allowance values, TR units, and sequencing error rates. Fig. 1A and 1B respectively show accuracy when the allowance parameter is set to 0% and 2%^1^. The accuracy of 2% allowance (Fig. 1B) is remarkably better than that of 0% allowance (Fig. 1A). In most cases, uTR outperformed RepeatMasker and TRF in terms of prediction accuracy, and this is especially true when mosaic TRs have three or more series of units. Prediction accuracy of uTR, RepeatMasker, and TRF tends to decrease as the sequencing error rate increases because sequencing errors obscure the original unit patterns and make prediction hard.

While prediction accuracy is essential in evaluating the usefulness of the tools, their computational performance was also measured using the same 48 datasets with 1000 mosaic TRs (Fig. 1C). Computational performance was evaluated using the Apple M1 Max processor (10 high-performance cores, clocked at 3.228 GHz) and 64 GB of main memory. On most of the 48 datasets, uTR was faster than RepeatMasker and TRF. Since the time required to verify that a mosaic TR pattern meets the allowance condition is a small constant, allowance differences do not affect computational performance. Higher error rates resulted in longer computation times presumably because more errors produced more candidate units with sequencing errors. Figure 1D shows the average length of each of the eight mosaic TRs, showing that processing longer mosaic TRs also needs longer computation time. Overall, uTR is more accurate and faster than RepeatMasker and TRF.

We now turn our attention to the ambiguity of the decomposition; i.e. a single string can be decomposed into several optimal mosaic TR patterns that minimize the (revised) penalty. For example, has two optimal decompositions:


AGAGAGGGAGGGAGAGAGGGAGGG

(AG)2(AGGG)2(AG)2(AGGG)2 and (AGAGAGGGAGGG)2

The above two have the same penalty of 14 (= 2 + 4 + 4 + 4 = 12 + 2). The source of the ambiguity is the duplication of subpattern (AG)2(AGGG)2. Although it is correct to select one of the two as the best one, this is a corner case because (AG) *i*(AGGG) *j*(AG) *k*(AGGG) *m* is the unique optimal answer when (AG) occurs two or more times (2≤i,k) while (AGGG) occurs three or more times (3≤j,m). When we generated the benchmark string datasets, we avoided such duplication of subpatterns as much as possible and eliminated the ambiguity of obtaining multiple correct decompositions. Put another way, each dataset was designed to have a single optimal decomposition and to be easily checked for consistency with one hidden pattern.

### 3.2 Detecting real mosaic tandem repeats with uTR

To demonstrate the practical application of uTR, we show that uTR could detect the following mosaic TRs in publicly available sequence data. The first two TRs are collected from patients with benign adult familial myoclonic epilepsy (BAFME), while the latter three are obtained from the human reference genome hg38.

- (ATTTT)221 (ATTTC)221 (ATTTT)82 is in the 4th intron of *SAND12* and is found in Patient II-1 of BAFME in family F6115 (see Supplementary Fig. S6 in Ishiura et al. 2018).
- (ATTTT)613 (ATTTC)320 (ATTTT)5 (ATTTC)130 is another type in the 4th intron of *SAND12* and is found in Patient II6 of BAFME in family F6906 (see Supplementary Fig. S6 in Ishiura et al. 2018).
- (AAAAG)11 in an intron of *RFC1* is located at chr4:39 348 425-39 348 483 in the human reference genome hg38. (AAGGG) expansions associated with CANVAS have been reported at the same locus (Cortese et al. 2019), but the sequence data is not publicly available for privacy protection reasons. Thus, we used the (AAAAG) expansion in the reference.
- (AAAG)6 (AG)11 (AAAG)20 is in an intron of *KAZN* and is at chr1:14 883 297-14 883 426 in reference hg38.
- (CTTTT)12 (CTTGT)3 (CTTTT)2 is in an intron of *ZNF37A* and is at chr10:38 112 731-38 112 826 in reference hg38.

uTR could identify all the mosaic TRs, while TRF detected the third one. A fasta file with the DNA sequences of the above mosaic TRs is available at https://github.com/morisUtokyo/uTR.

### 3.3 Merit of using non-self-overlapping units

**Figure 2.**
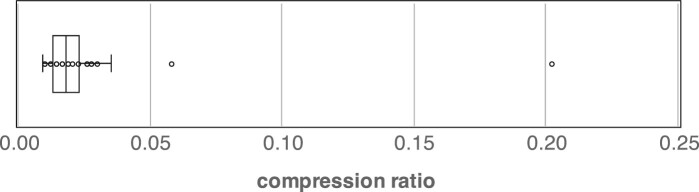
Compression ratio distribution: the boxplot shows the first, second, and third quartiles.

Here, we show that using non-self-duplicating units is quite effective in reducing the number of units to be considered. To quantify the benefits, let *W_all_* be the set of all unique substrings in string *S* of length *θ* or less (e.g. *θ * = 20), and *W_nsop_* be the set of all non-self-overlapping substrings in *W_all_*. The ratio of the sizes of *W_nsop_* and *W_all_*, $\left|W_{\mathrm{nsop}}|/|W_{\mathrm{all}}\right|$, is called the *compression ratio* by non-self-overlapping. To measure the compression ratio, we examined the 48 datasets that were used to measure the prediction accuracy in Fig. 1. Figure 2 presents the distribution of the compression ratios of the 48 files, and most compression ratios were 5% or less, highlighting a remarkable reduction in the number of units.

## 4 Discussion

It is an open question to understand the computational complexity of calculating a pair of unit set *U* and decomposition *D* that minimizes $\sum_{u\in U}(|u|+o(u))+\sum_{s\in D,s\notin U}|s|$. We speculate on its intractability. This is because we studied a restricted case when *U* is fixed, asked whether there is a decomposition of *S* for which the penalty $\sum_{u\in U}(|u|+o(u))$ is *T* or less for a given constant *T*, which is called the unit coding problem, and we proved its NP-completeness (Supplementary Appendix).

Our program uTR estimates a mosaic TR pattern for an input DNA string, but the pattern and the string may have a number of mismatches because of variants in units. Resolution of this problem is medically important because for example, some variants in minisatellite units have been reported to be associated with several brain diseases (Cortese et al. 2019; Course et al. 2020, 2021; De Roeck et al. 2018; Song et al. 2018). To identify unit variants in a minisatellite that has the representative form (u)n, one can compare the given string and the concatenation of *n* copies of *u* by using a dynamic programming alignment algorithm, and find an optimal concatenation of *u’*s variants (*u_i_* for i=1,…,n). This concept can be generalized to mosaic TRs, and a program for this purpose is available at: https://github.com/morisUtokyo/vTR.

## 5 Conclusion

Expanded mosaic TRs have attracted a great deal of attention because they are relevant to a number of disorders (Depienne and Mandel 2021). Most mosaic TRs require a long read sequence to cover the entire sequence, but it is difficult to automatically characterize the sequence configurations of mosaic TRs, so they have been analyzed by manual inspection and remain largely unknown. To resolve this problem, we proposed how to measure the goodness of selecting units in mosaic TRs and developed an efficient algorithm for calculating a better unit set according to the measurement. After selecting units, it is tractable to compute an optimal concatenation of the units that partially matches the input string with the minimum Levenshtein distance in the presence of sequencing errors (Myers and Miller 1989). We demonstrated the high accuracy and computational efficiency of our program using synthetic benchmark data modeling typical mosaic TRs of human genomes.

The next goal is to calculate the distribution of mosaic TRs by applying this program to long reads collected from individual genomes from a healthy population. It will then be important to apply the program to long reads from affected individuals and to compare the mosaic TRs to those of a healthy population to determine which repeating units are significantly expanded and cause disease. We expect the program to be used to assist in this type of discovery, which to date has been performed manually.

## Supplementary Material

btad185_supplementary_data

## Notes

1 Using a larger allowance (e.g., 3%) produced a similar tendency.
