# Supplementary material for: Decomposing mosaic tandem repeats accurately from long reads
Source: Bioinformatics. 2023 Apr 11;39(4):btad185. doi: 10.1093/bioinformatics/btad185 (PMC10118999; doi:10.1093/bioinformatics/btad185)
Supplement: btad185_supplementary_data [file btad185_supplementary_data.pdf]

Appendix

Proof of NP-completeness of UEP

Theorem 1. *The unit encoding problem (UEP) is NP-complete. Given a set of alphabets Σ, a string $S \in \Sigma^*$, a set of strings $U \subset \Sigma^*$, and an integer T, is there a decomposition $S = s_1 \cdots s_k$ such that all elements s_i appear in U and $\sum_{s \in \{s_1, \cdots, s_k\}} \{|s| + o(s)\} \leq T$?*

To avoid double counting of the same substring appearing in multiple positions of D, $\{s_1, \cdots, s_k\}$ represents a non-redundant set of the decomposition. According to this lemma, it is NP-hard to calculate an optimal decomposition that minimizes $\sum_{s \in \{s_1, \cdots, s_k\}} \{|s| + o(s)\}$. The problem may become harder when U is not provided, but we do not have an answer to the intractability of this extended situation.

We show this lemma by giving a polynomial time reduction from the vertex covering problem, a well-known NP-complete problem: for a graph $G = (V, E)$ and an integer K, check whether or not there is a subset $V' \subset V$ of size K or smaller such that for each edge (u, v), either u or v is in V' (Karp, 1972).

Let $G = (V, E)$ and K denote an instance of the vertex covering. For convenience, we express V as a set of integers $\{1, \cdots, N\}$ and an edge as a pair of integers (i, j) such that $i < j$. In addition, we define the maximum degree of G as A. We assume $A > 1$ because the vertex covering problem is trivial if $A \leq 1$. We convert G and K to an instance of the UEP (Σ, S, U, T) to answer the vertex covering problem of G and K.

We will illustrate the reduction using a simple working example: the nodes are $V = \{1, 2, 3, 4\}$, the edges are $E = \{(1, 2), (2, 3), (3, 4)\}$, and K is set to 2. The answer in this instance is "yes," because $\{1, 3\}$ is one of the optimal vertex coverings.

In general, we can use a so-called adjacency list to represent a graph by a string. Formally, we treat each edge $(i, j) \in E$ as a character in Σ, encode each node $i \in V$ by a concatenation of all edges with i as one node, which are of the form $(i, -)$ or $(-, i)$, and denote the concatenation by S_i. For examples, $2 \in V$ is encoded by $(2, 3)(1, 2)$. By merging all S_is, we obtain the adjacency list $S \in \Sigma^*$ of G. For example, the adjacency list S of the graph is

$$S = \overbrace{(1,2)}^{S_1}\overbrace{(2,3)(1,2)}^{S_2}\overbrace{(2,3)(3,4)}^{S_3}\overbrace{(3,4)}^{S_4}. \quad (1)$$

We represent a vertex covering V' by decomposing S into S_is and subsequently S_i into single letters if node i is a member of V'. In other words, we connect a covering V' of G and a decomposition D of S via the rule:

$$i \in V' \iff D \text{ decomposes } S_i \text{ into single letters.} \quad (2)$$

In the running example, we represent an optimal covering, $\{1, 3\}$, as

$$D = \overbrace{(1,2)}^{1 \in V'} \cdot (2,3)(1,2) \cdot \overbrace{(2,3) \cdot (3,4)}^{3 \in V'} \cdot (3,4). \quad (3)$$

Here, we use "$\cdot$" as the symbol for decomposition. The penalty of D is 10 because each of $(1, 2)$, $(2, 3)(1, 2)$, and $(2, 3)$ occurs once, $(3, 4)$ occurs twice, and the penalty is $(1 + 1) + (2 + 1) + (1 + 1) + (1 + 2) = 10$.

Although the adjacency list representation seems to provide a way to convert vertex covering to UEP, there are three issues to be solved.

First, there is no guarantee that optimal decompositions of S correspond to optimal coverings of G. Specifically, one of the optimal decompositions of S is

$$\overbrace{(1,2)}^{S_1}\overbrace{(2,3)\cdot(1,2)}^{S_2}\overbrace{(2,3)\cdot(3,4)}^{S_3}\overbrace{(3,4)}^{S_4}$$

with a penalty of seven because $(1, 2)(2, 3)$ occurs twice, $(3, 4)(3, 4)$ once, and the penalty is $(2 + 2) + (2 + 1) = 7$. But this decomposition is inconsistent with nodes grouped by the overbraces in Equation (1), and no coverings correspond to it.

Second, the decompositions for a non vertex covering and an optimal covering can have the same penalty. For example, suppose that V' is $\{1\}$. V' is not even a covering, and the corresponding decomposition is

$$\overbrace{(1,2)}^{1 \in V'} \cdot (2,3)(1,2) \cdot (2,3)(3,4) \cdot (3,4).$$

This decomposition and that of (3) share the same penalty, ten because all substrings occur once, and the penalty is $(1 + 1) + (2 + 1) + (2 + 1) + (1 + 1) = 10$.

Finally, we cannot distinguish optimal coverings from non-optimal coverings. When V' is a non-optimal vertex covering $\{1, 3, 4\}$, the decomposition is again the same as that in (3).

Considering these issues, it is necessary to refine this adjacency list representation. As the refinement includes complicated techniques, we provide the motivations, conversions, and explanations of why they work.

First, for prohibiting decompositions violating the overbraces in Equation (1), we introduce a set of units U such that all substrings of D are in U, We introduce two new characters `D` and `F` to simplify the definition of U.

Second, we ensure that the penalty is much smaller for a valid decomposition built from a covering than for an invalid decomposition. For this purpose, we intuitively append all of the edges at the tail of S:

$$S = \overbrace{(1,2)(2,3)(2,1)(3,2)(3,4)(3,4)}^{\text{the adjacency list of } G} \cdot \overbrace{(1,2)\cdot(2,3)\cdot(3,4)}^{\text{covering checkers}} \quad (4)$$

The penalty of an appended edge, say $(2, 3)$, increases by 1 if V' is a covering because $(2, 3)$ should already appear as a substring in D (see $(2, 3)(3, 4)$ in Equation (3) for example). In contrast, it increases by 2 if V' fails to include either 2 or 3. Therefore, the appended edge-characters work as "covering checkers" in that they increase the penalty every time V' fails to cover an edge. To enlarge the incurred penalty sufficiently, we define a new auxiliary character `E` and replace each edge-character, such as $(2, 3)$, with a long string $\mathtt{E} \cdots \mathtt{E}(2, 3)\mathtt{E} \cdots \mathtt{E}$.

The final aim is to link the penalties of decompositions with the sizes of the corresponding coverings, $|V'|$. With these motivations in mind, we newly define the alphabet Σ, the set of units U, the string S, and the threshold parameter T from scratch. To begin with, we set $U = \emptyset$ and $\Sigma = \{(i, j) \mid (i, j) \in E\}$. We then introduce a new punctuation mark, denoted by `D`, and add `D` to Σ. We also treat `D` as a single-character string and add it into U. As long as no units contain `D` except for `D` itself, any decomposition D of S isolates all occurrences of `D` as elements of D.

We will then ensure that all of the decompositions of S_is have the same penalty and are independent of each other, let A be the maximum degree of G and N be the number of nodes in the graph. We then extend the graph such that Σ has A characters of the form of $(i, -)$ or $(-, i)$ for each i. In the working example, since $A = 2$, the characters corresponding to the edges are $\{(1, 2), \underline{(1, 5)}, (2, 3), (3, 4), \underline{(4, 5)}\}$. We underline the newly introduced characters. In general, if the degree of i, $d(i)$, is less than A, we add $(i, N + 1), \cdots, (i, N + A - d(i))$ to Σ, where $N + 1, \cdots, N + A - d(i)$ are new nodes.

To make the penalties of a decomposition of S_i independent of each other, any pairs of S_is should not share any substrings in U. For this

picture(0,0)(-35,0)(1,0)30 (0,35)(0,-1)30 picture picture(0,0)(35,0)(-1,0)30 (0,35)(0,-1)30 picture

purpose, let L be a sufficiently large integer, e.g., $4|E| + (K + 3)N$. Then, we distinguish all edges by associating node i of edge (i, j) with

$$\mathtt{E}^{L+2i}(i, j)\mathtt{E}^{L+2N-2i+1},$$

which we denote by $P_i(i, j)$. For ease of understanding, we illustrate $P_i(i, j)$ and $P_j(i, j)$:

$$P_i(i,j) = \overbrace{\mathtt{EEE}\cdots\mathtt{EEE}}^{L+2i}(i,j)\overbrace{\mathtt{EEEEE}\cdots\mathtt{EEEEE}}^{L+2N-2i+1}$$
$$P_j(i,j) = \overbrace{\mathtt{EEEEE}\cdots\mathtt{EEEEE}}^{L+2j}(i,j)\overbrace{\mathtt{EEE}\cdots\mathtt{EEE}}^{L+2N-2j+1}$$

It is clear that $P_i(i, j)$s contain their own edge characters (i, j), are of the same length $2(L + N + 1)$, and have unique tags ($\mathtt{E}^{L+2i}$) at the left side of (i, j).

Then, we introduce another new punctuation character $\mathtt{F}$ to Σ and concatenate all $P_i(i, j)$s and $P_i(j, i)$s, separating them with $\mathtt{F}$:

$$S_i = \prod_{(i,j)} P_i(i,j)\mathtt{F} \prod_{(j,i)} P_i(j,i)\mathtt{F}$$

as illustrated by S_1 and S_3 in Figure3. As designed, all S_is now have the same length, $A(2(L + N + 1) + 1)$ (1 for $\mathtt{F}$), and all $P_i(i, j)$s and $P_j(i, j)$s are distinct. We add all $P_i(i, j)$s and all S_is to U.

Finally, it is necessary to implement the "covering checker" as illustrated in (4) so as to validate that the corresponding subset of nodes $V' \subset V$ is indeed a covering. For this purpose, we take an approach of computing the penalty of the optimal decomposition of $S_{i,j} = \mathtt{E}^{2L}(i, j)\mathtt{E}^{2L}$. Suppose we have decomposed $S_{i,j}$ into either of the following:

$$\underbrace{\mathtt{E}^{L-2i}}_{\text{added to }U} \cdot \underbrace{\mathtt{E}^{L+2i}(i,j)\mathtt{E}^{L+2N-2i+1}}_{P_i(i,j)} \cdot \underbrace{\mathtt{E}^{L-2N+2i-1}}_{\text{added to }U} \quad (5)$$

$$\underbrace{\mathtt{E}^{L-2j}}_{\text{added to }U} \cdot \underbrace{\mathtt{E}^{L+2j}(i,j)\mathtt{E}^{L+2N-2j+1}}_{P_j(i,j)} \cdot \underbrace{\mathtt{E}^{L-2N+2j-1}}_{\text{added to }U} \quad (6)$$

We can compute the penalties of $\mathtt{E}^{L-2i}$, $\mathtt{E}^{L-2N+2i-1}$, $\mathtt{E}^{L-2j}$, and $\mathtt{E}^{L-2N+2j-1}$ with ease by adding all of these patterns joined by $\mathtt{D}$, $\prod_{i=1}^{2N}(\mathtt{E}^{L-i}\mathtt{D})$, at the head of S (see "auxiliary domain" in Fig(3)). This is because, as S should be partitioned by $\mathtt{D}$, each of $\mathtt{E}^{L-1}, \cdots, \mathtt{E}^{L-2N}$ occurs at least once in any decomposition.

Now, the penalty of $S_{i,j}$ increases by 3 if either $P_i(i, j)$ or $P_j(i, j)$ is present in a decomposition and by greater than 3 otherwise. In addition, there are no decompositions of $S_{i,j}$other than (5) or (6) for U, because (i, j) appears only in $P_i(i, j)$, $P_j(i, j)$, S_i, and S_j. Since S_i and S_j have at least one $\mathtt{F}$, they cannot be any substrings of $S_{i,j}$.

Here, we summarize our argument so far. First, we convert vertices and edges in the graph as follows:

$$S_i = \prod_{(i,j)} P_i(i,j)\mathtt{F} \prod_{(j,i)} P_i(j,i)\mathtt{F} \quad \text{for each } i \in V$$
$$\text{where } P_i(i,j) = \mathtt{E}^{L+2i}(i,j)\mathtt{E}^{L+2N-2i+1}$$
$$S_{i,j} = \mathtt{E}^{2L}(i,j)\mathtt{E}^{2L} \quad \text{for each } (i,j) \in E,$$

Then, we define S and U as

$$S = \mathtt{FD}\overbrace{\prod_{i=1}^{2N}(\mathtt{E}^{L-i}\mathtt{D})}^{\text{aux. domain}}\overbrace{\prod_i(S_i\mathtt{D})}^{\text{the adj. list}}\overbrace{\prod_{(i,j)\in E}(S_{i,j}\mathtt{D})}^{\text{covering checkers}}$$
$$U = \{\mathtt{D},\mathtt{F}\} \cup \{S_i \mid i = 1, \cdots, N\} \cup$$
$$\{P_i(i,j), P_j(i,j) \mid (i,j) \in \Sigma\} \cup$$
$$\{\mathtt{E}^{L-i} \mid i = 1, \cdots, 2N\}$$

Figure 3 shows the conversion of the running example.

The correspondence between a covering V' and a decomposition is:

$$i \in V' \iff S_i \text{ is split into } P_i(i,-)\cdot\mathtt{F}\cdot\ldots\cdot\mathtt{F}\cdot P_i(-,i)\cdot\mathtt{F} \quad (7)$$

That is, D decomposes S_i into A pieces of $P_i(i, -)$ or $P_i(-, i)$ and A pieces of $\mathtt{F}$ if i is in V' and does not decompose S_i at all if $i \notin V'$.

What is the penalty of this decomposition if V' is a vertex covering with K or fewer vertices? The leading $\mathtt{F}\cdot\mathtt{D}$ incurs $(1 + 1) + (1 + 1) = 4$ penalty. For the subsequent string $\prod_i \mathtt{E}^{L-i}\mathtt{D}$, as $\mathtt{D}$ exists uniquely in U, there is only one way to decompose:$\mathtt{E}^{L-1}\cdot\mathtt{D}\cdot\ldots\cdot\mathtt{E}^{L-2N}\cdot\mathtt{D}$. Given there are $2N$ $\mathtt{D}$s, the penalty is $(2L - 2N + 3)N$ in total.

To calculate the penalty corresponding to nodes, we divide the case according to whether it is in the covering. Recall that the size of S_i is $A(2(L + N + 1) + 1)$, and let $\hat{L}$ denote the size $|S_i|$.

- If $i \in V'$, S_i is decomposed into A pieces of $P_i(i, -)$ (or $P_i(-, i)$) and $\mathtt{F}$, and the penalty is $\hat{L} + A$, where the A is the number of occurrences of S_i.
- If $i \notin V'$, S_i is not decomposed, and the penalty is $|S_i| + 1 = \hat{L} + 1$.

In total, the penalty is

$$|V'|(\hat{L} + A) + (N - |V'|)(\hat{L} + 1) = N\hat{L} + N + (A - 1)|V'|$$
$$\le N\hat{L} + N + (A - 1)K.$$

For $\prod_{(i,j)\in E}(S_{i,j}\mathtt{D})$, because V' is a covering, the penalty of each $S_{i,j}$ is 3 as shown in (5) and (6). As there is an additional $\mathtt{D}$ for each $S_{i,j}$, the penalty is 4, summing up to $4|E|$ in total. By aggregating these penalties, we have:

$$4 + (2L - 2N + 3)N + \hat{L}N + N + (A - 1)K + 4|E|,$$

as an upper bound of the penalty. Assigning the above bound to T provides a positive answer to the unit encoding problem.

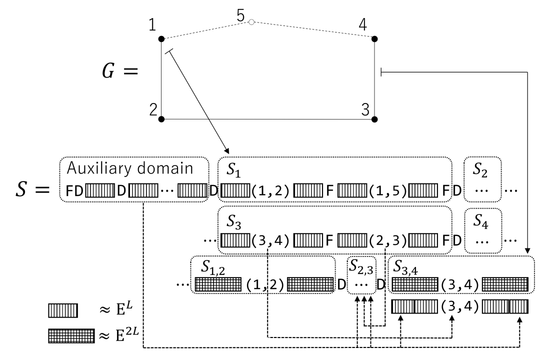

Fig. 3. A simple example of reduction from a vertex covering problem to UEP. The graph is $V = \{1, 2, 3, 4\}$ and $E = \{(1, 2), (2, 3), (3, 4)\}$. Boxes with stripes represent strings of $\mathtt{E}$ with the length of approximately L, while boxes with checks represent those with a length of approximately $2L$.

picture(0,0)(-35,0)(1,0)30 (0,-35)(0,1)30 picture picture(0,0)(35,0)(-1,0)30 (0,-35)(0,1)30 picture

To complete the proof, it remains to confirm the reverse direction; Given a decomposition D of S with a penalty of no greater than T, can we construct a vertex covering with the size at most K?

First, because the character D uniquely exists in U, D splits S at least by D. In addition, D must decompose $\prod_i (\mathtt{E}^{L-i}\mathtt{D})$ into $\mathtt{E}^{L-1} \cdot \ldots \cdot \mathtt{E}^{L-2N}$, the penalty of which is $(2L-2N+3)N$.

Considering how we build S_i, we have only two ways to decompose S_i; to partition it by F or to leave it as it is. These decompositions incur $\hat{L}+A$ penalty and $\hat{L}+1$ penalty, respectively. Let M be the set of vertices i such that D splits S_i by F. Then, the penalty would be:

$$|M|(\hat{L}+A)+(N-|M|)(\hat{L}+1)=N\hat{L}+N+(A-1)|M|$$

Summing the penalties of D, F, S_i, and the auxiliary domain, the total penalty of edges is

$$T' = 4+(2L-2N+3)N+N\hat{L}+N+(A-1)|M|+|E|$$

As we assume that the total penalty is T or less, the remaining penalty is $T-T' = 3|E|+(A-1)K-(A-1)|M|\ (\leq L)$.

We can only use (5) and (6) to decompose $S_{i,j}$ into either S_i or S_j by F. This is because, if otherwise, there would be at least one $S_{i,j}$ incurring an additional $|P_i(i,j)|+1 = |P_j(i,j)|+1 (> L)$ penalty. Thus, M is a vertex covering of G, and the penalty is three for each $S_{i,j}$. The total penalty becomes $3|E|$. As the overall penalty is at most T, we have $T-(T'+3|E|) = (A-1)(K-|M|) \geq 0$, or equivalently, $|M| \leq K$. Thus, M is a vertex covering of G less than or equal to K. $\square$
